# Supplementary material for: Additively manufacturable micro-mechanical logic gates
Source: Nat Commun. 2019 Feb 20;10:882. doi: 10.1038/s41467-019-08678-0 (PMC6382908; doi:10.1038/s41467-019-08678-0)
Supplement: Supplementary file 1 — Supplementary Information [file 41467_2019_8678_MOESM1_ESM.pdf]

Supplementary Information for
"Additively Manufacturable Micro-Mechanical Logic Gates"

Song, et al.

Supplementary Table 1. Bi-stable flexure mechanism designs and their buckling mode shapes.

	Design A	Design B	Design C	Design D	Design E	Design F
Undeformed Shape:	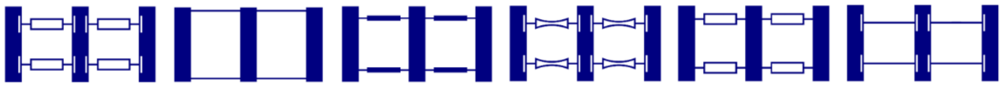					
1st buckling mode shape:	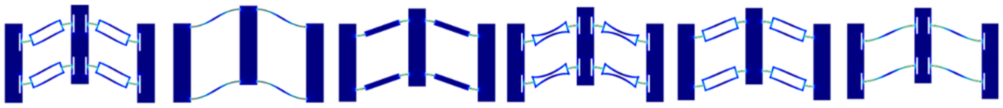					
1st buckling threshold:	h_1=0.015L	h_1=0.002L	h_1=0.002L	h_1=0.013L	h_1=0.006L	h_1=0.010L
2nd buckling mode shape:	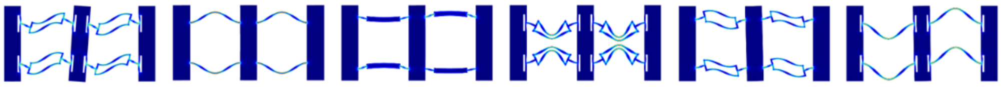					
2nd buckling threshold:	h_2=0.163L	h_2=0.008L	h_2=0.030L	h_2=0.113L	h_2=0.070L	h_2=0.041L

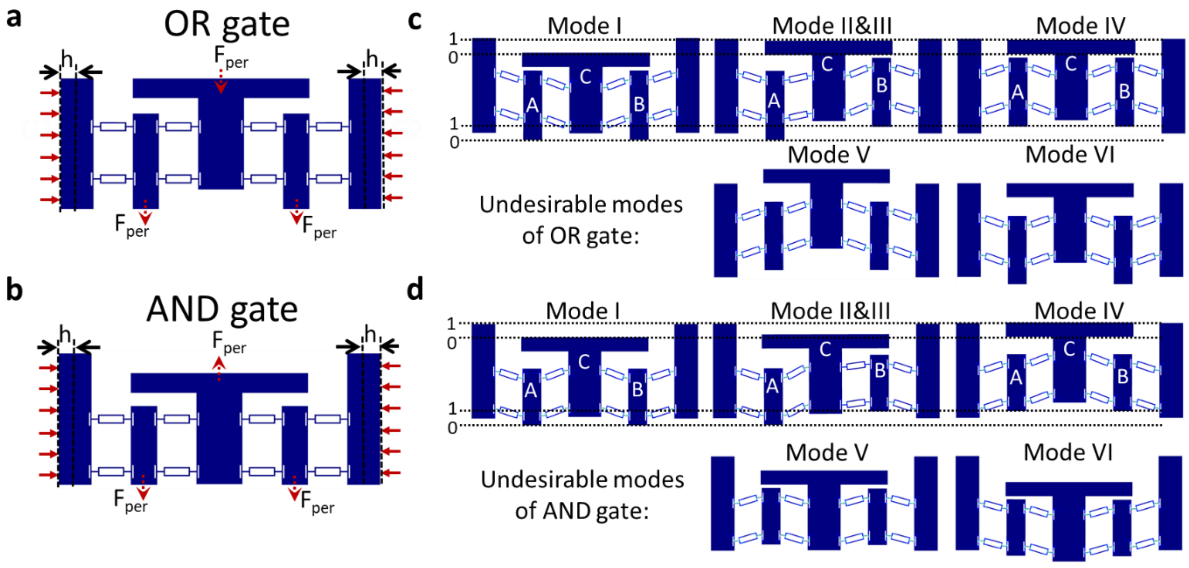

Supplementary Figure 1. Design of the OR gate and the AND gate. a) Undeformed OR gate subject to the initial compression and perturbations. b) Undeformed AND gate subject to the initial compression and perturbations. c) Buckling mode shapes of the OR gate. d) Buckling mode shapes of the AND gate.

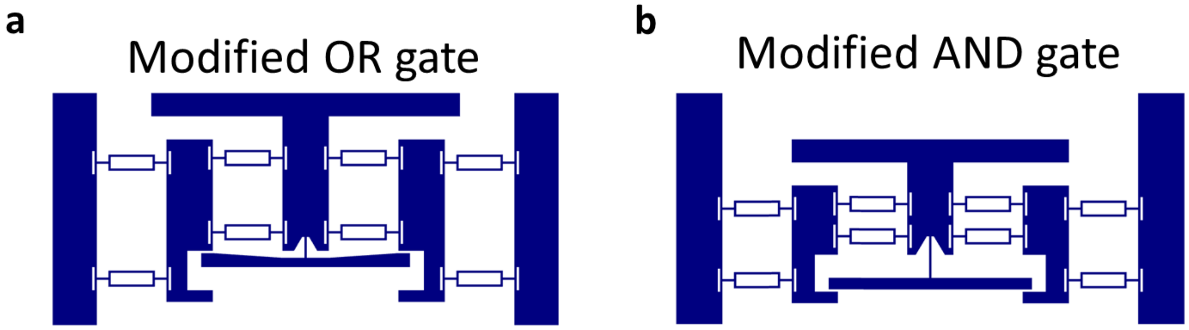

Supplementary Figure 2. Modified logic gate designs. a) Modified OR gate design with an additional rotational rigid bar and mechanical stops. b) Modified AND gate design with an additional rotational rigid bar and mechanical stops.

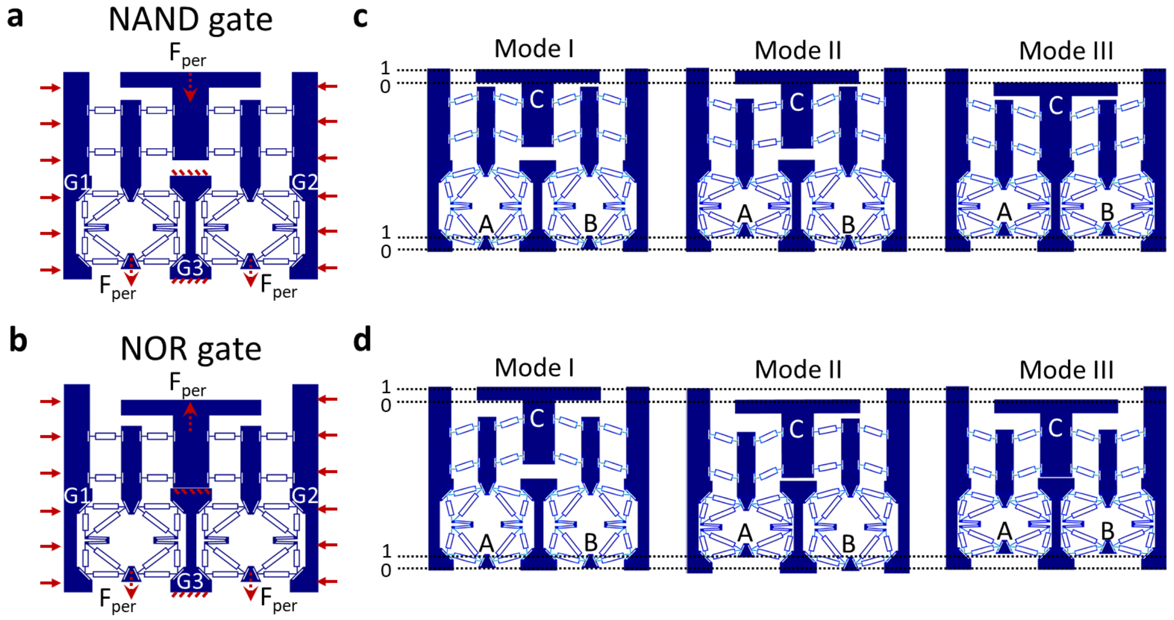

Supplementary Figure 3. Design of the NAND gate and the NOR gate. a) Undeformed NAND gate subject to the initial compression and perturbations. b) Undeformed NOR gate subject to the initial compression and perturbations. c) NAND gate at different logic states. d) NOR gate at different logic states.

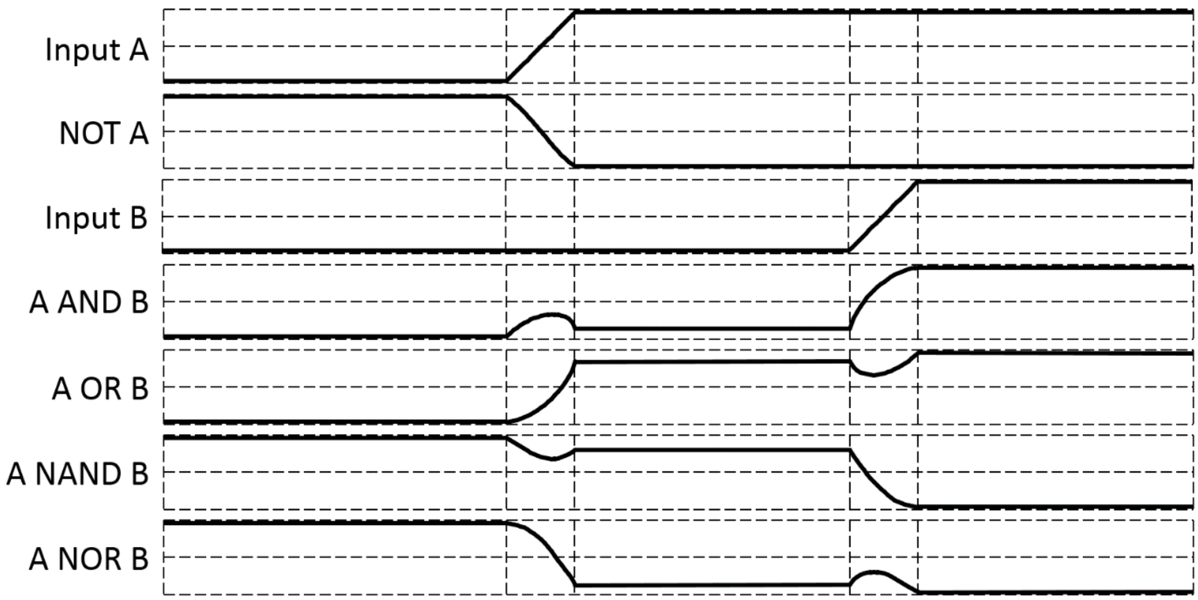

Supplementary Figure 4. Quasi-static timing diagram of the outputs of the proposed mechanical logic gates when the inputs (A, B) transition from (0, 0) to (1, 0) to (1, 1).

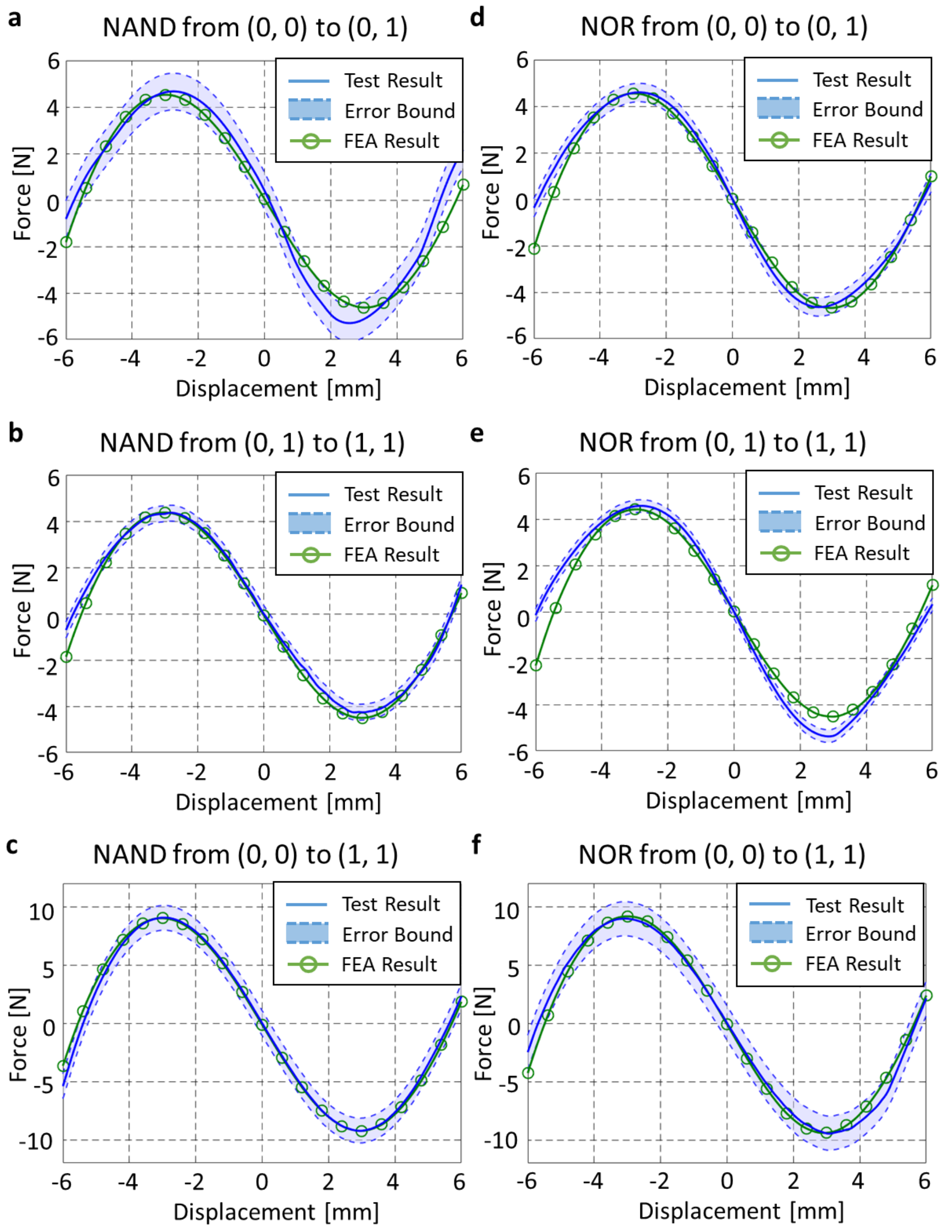

Supplementary Figure 5. FEA results of the macroscale mechanical NAND and NOR compared against experimental data with 95% error bounds. a) Force on one of NAND's inputs as the input transitions from 0 to 1 while the other input maintains 0. b) Force on one of NAND's inputs as the input transitions from 0 to 1 while the other input maintains 1. c) Force on both inputs of NAND as the inputs transition from 0 to 1. d) Force on one of NOR's inputs as the input transitions from 0 to 1 while the other input maintains 0. e) Force on one of NOR's inputs as the input transitions from 0 to 1 while the other input maintains 1. f) Force on both inputs of NOR as the inputs transition from 0 to 1.

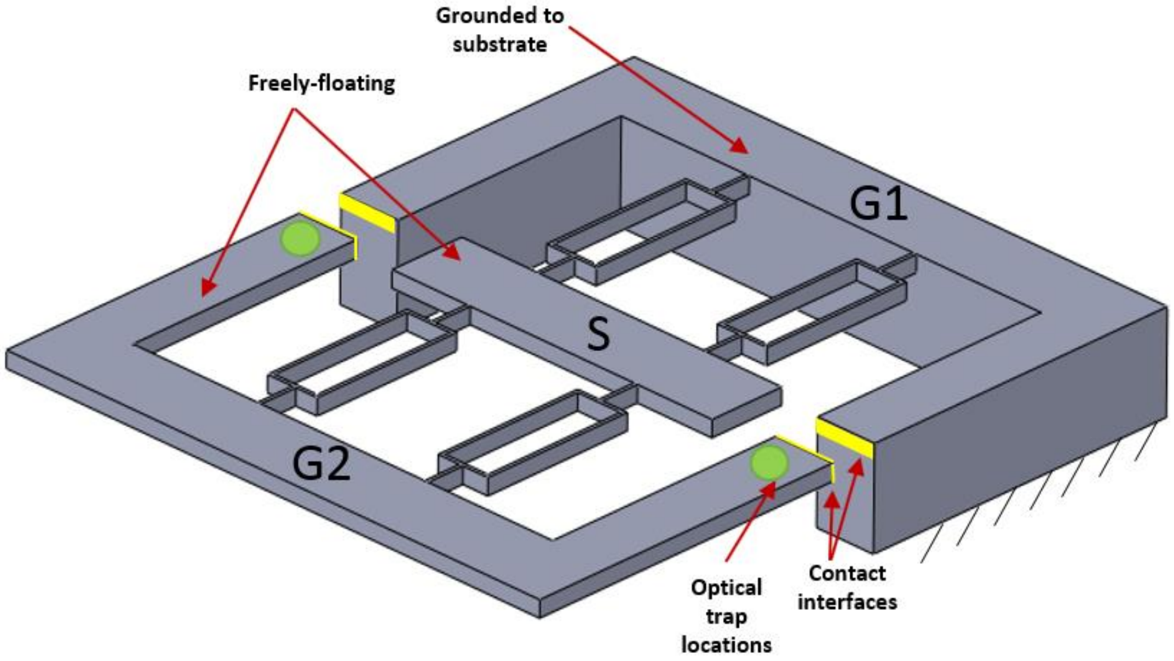

Supplementary Figure 6. 3D CAD representation of the bi-stable buckling flexure element printed via 2PS.

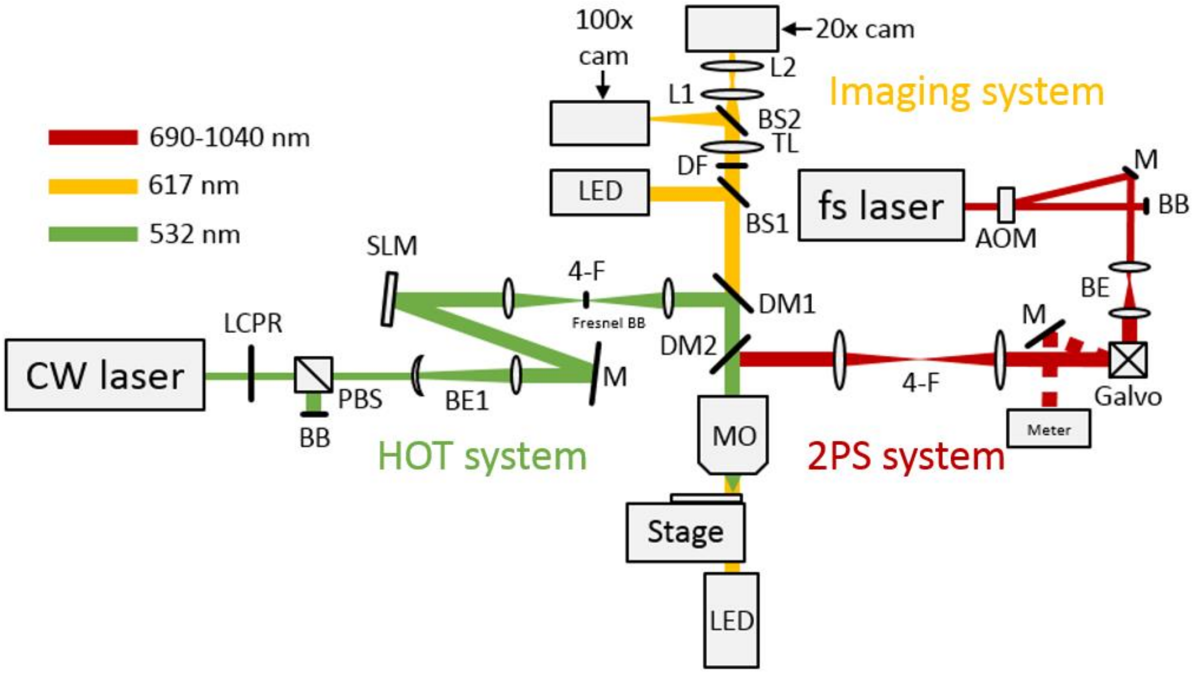

Supplementary Figure 7. Experimental setup combining the 2PS and HOT approaches.

Supplementary Note 1

The AND gate and the OR gate both consist of two buckled flexure mechanisms connected in series, as shown in Supplementary Figure 1. When compressed horizontally under the perturbations shown in Supplementary Figure 1a and b, the structures deformed into one of their first buckling mode shapes, labeled as Mode I in Supplementary Figure 1c and d, where the two inputs (A, B) are (0, 0). As the two inputs varied among different logic states (i.e., Mode I, Mode II&II, and Mode IV), both gates generated the correct result on the output C within a 10% error. Note that if a strong external disturbance is applied on the rigid body "C", it is possible that the "undesirable" mode shapes (i.e., Mode V and Mode VI) may occur and the logic gates may generate wrong outputs as shown in Supplementary Figure 1c and d. This issue can be solved by adding mechanical stops and attaching a rotational rigid bar to the body "C" (Supplementary Figure 2). In such a way, the position of the rigid body "C" can be strictly constrained, and the undesirable mode shapes can be eliminated.

The designs of the NAND and NOR gates are shown in Supplementary Figure 3. To initiate the buckling of the structures, the two rigid bodies G1 and G2 were compressed inwards under the perturbations shown in Supplementary Figure 3a and b, while the rigid body G3 was fixed. The geometric configurations of NAND and NOR at different logic states are shown in Supplementary Figure 3c and d. The quasi-static timing diagrams of the input(s) and the output of all designed logic gates were obtained using COMSOL as shown in Supplementary Figure 4. Two macroscale logic gates, a NAND and a NOR, are fabricated using a Fused Deposition Modeling (FDM) 3D-printer. The force-displacement relationships of the macroscale NAND and NOR's input(s) were experimentally measured, and the results were compared against the FEA results (Supplementary Figure 5).
