# Supplementary material for: Additively manufacturable micro-mechanical logic gates
Source: Nat Commun. 2019 Feb 20;10:882. doi: 10.1038/s41467-019-08678-0 (PMC6382908; doi:10.1038/s41467-019-08678-0)
Supplement: Supplementary file 3 — Description of Additional Supplementary Files [file 41467_2019_8678_MOESM3_ESM.pdf]

Description of Additional Supplementary Files

Supplementary Movie 1. Actuation of the macroscale NAND and NOR logic gates as well as fabrication and testing of the microscale bi-stable mechanism.
